# Is Colombia a Violent Country?

**Published:** 2017-03-30

**Authors:** Rodrigo Guerrero, Andrés Fandiño-Losada

**Affiliations:** 1 Instituto CISALVA, Escuela de Salud Publica, Facultad de Salud, Universidad del Valle, Cali, Colombia.

"In Colombia, violence doesn't disappear-it transforms," the director of the National Institute for Legal Medicine and Forensic Sciences said recently ^1^ He noted the long history of political violence that has persisted in Colombia despite several peace processes, social violence in the form of children dying from malnutrition, child abuse cases numbering more than 18,000 by October of last year, acid attacks on women, etc. The recent cruel killing of an eight-year-old girl in Bogota is yet another manifestation of the multifaceted violence that exists in Colombia, and it has raised the question of whether Colombia is truly an exceptionally violent country.

The World Health Organization (WHO) defines violence according to three categories: self- directed, collective and interpersonal. Self-directed violence includes suicide; collective violence includes economic, political and social violence; and interpersonal violence includes domestic abuse against children, women or the elderly as well as violence between acquaintances or strangers. In Eurozone countries, self-directed violence is the most common type, while interpersonal violence dominates in the Americas ^2^. The latter category is measured in injuries and homicides, information about which can be easily obtained. According to the WHO, a homicide is a death resulting from the intentional use of physical force. Based on this definition, there are two indispensable criteria for an act to be considered a homicide: the intent to kill and the use of physical force.

**Figure 1:**
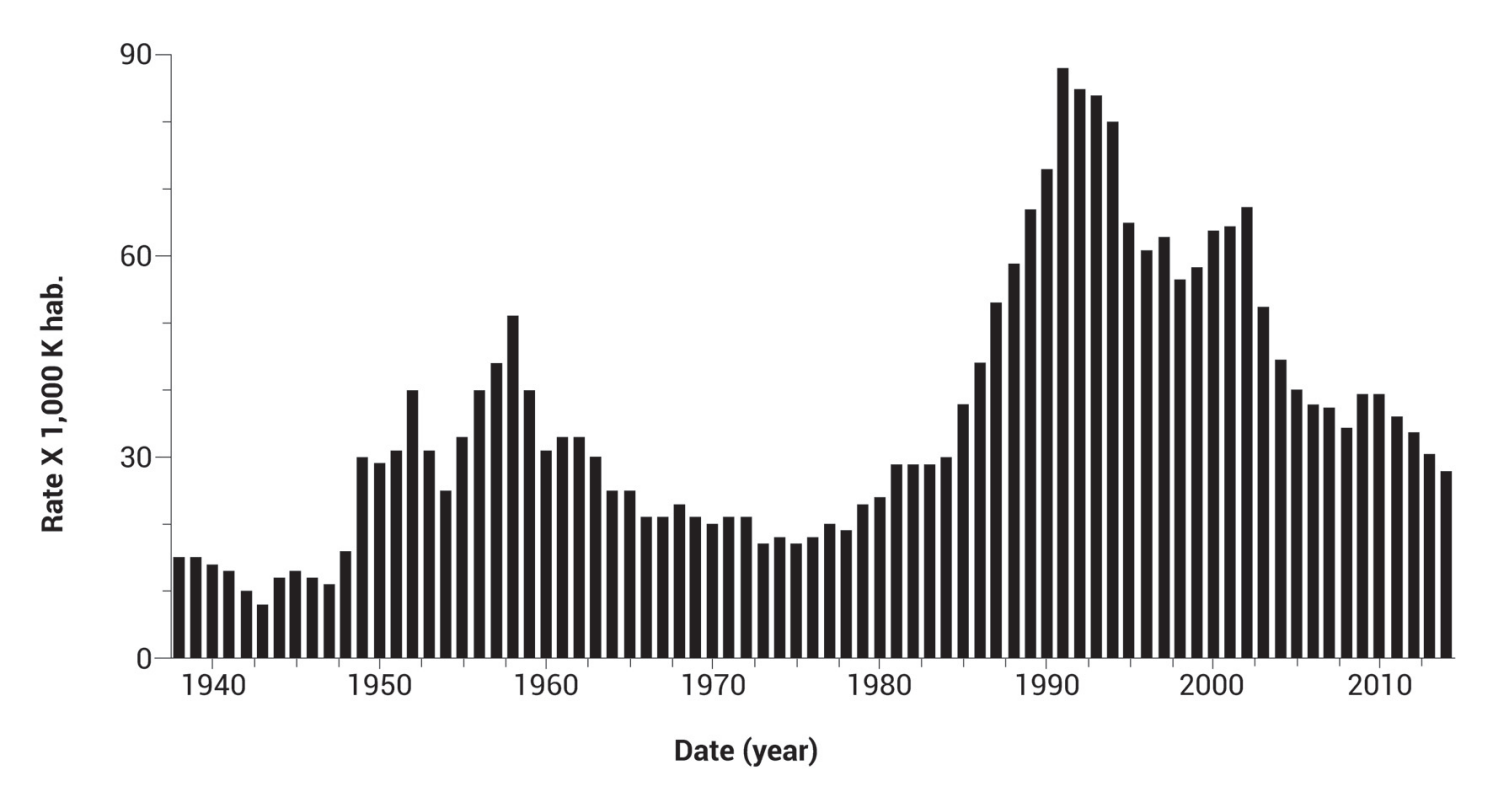
Homicide Rates in Colombia, 1938 to 2014. Prepared by: CISALVA Institute of the Universidad del Valle, Colombia. Sources of Data: National Institute of Forensic Medicine and Forensic Sciences / Group National Reference Center on Violence / Information System Network of Disappeared and Corpses / National Information System of Indirect Statistics. Rates calculated based on the population projection DANE 2005-2020. References: ^-^ ^3^,^-^ ^5^

Despite the wide variety of ways in which violence can occur, when we speak of cities or countries that are generally deemed violent, we are usually referring to the homicide rate per 100,000 inhabitants. Let us see how, based on this indicator, Colombia compares. A historical view can help us to understand the situation. When Colombia's National Statistics Department began publishing homicide statistics in 1938, the country had a rate of 16 homicides per 100,000 inhabitants (Fig. 1). This rate increased in the '50s and ranged between 20 and 30 per 100,000 up until the '80s, when it saw another rapid rise that would last through the early '90s. Since then, the rate has been declining.

Table 1 shows homicide rates for various countries in the Americas in 2013. One can see that Honduras, Jamaica and Guatemala had higher homicide rates than Colombia. Belize, the Dominican Republic, Brazil, Panama and Ecuador had lower homicide rates than Colombia. The rates in Nicaragua, Paraguay, Uruguay, Peru and Chile were lower still, in the single digits.^6^ ^,^ ^7^ In 2015, Colombia's homicide rate was 17 per 100,000 inhabitants, according to the INMLCF.^8^ It was still over 10, which is the threshold above which the WHO considers homicide to be an endemic problem.^9^

**Table 1:** Homicide Rates in the Americas in 2013

Country	Cases	Rate*
Honduras	6,431	75.2
Jamaica	1,200	44.2
Guatemala	5,253	34.0
Colombia	15,234	32.3
Belize	99	28.3
Dominican Republic	1,978	19.3
Brazil	57,045	17.5
Panama	666	17.3
Ecuador	1,723	10.9
Nicaragua	594	9.8
Paraguay	666	9.8
Uruguay	260	7.7
Peru	2,013	6.6
Chile	481	2.7
El Salvador	2,499	n.d.
Bolivia	938	n.d.

n.d. = no data.

*Rates per 100,000 inhabitants.

Created by the CISALVA Institute of the Universidad del Valle, Colombia.

Sources: Proyecto SES/Sub Unidades Técnicas Nacionales in participating countries (2014).^7^ No consolidated data are available for Argentina, Costa Rica, Guyana and Mexico for 2013.

Colombia is certainly a violent country compared to many of its neighbors in the Americas, a region that is considered one of the most violent in the world ^10^. The question is, why?

Colombia has lived in a permanent state of armed conflict; it endured more than 10 civil wars in the 19^th^ century and the Thousand Days' War in the early 20^th^ century. These were immediately followed by irregular political conflicts in various rural areas and culminated in the assassination of the liberal presidential candidate Jorge Eliécer Gaitán in 1948. At that point, the country entered a phase known as "The Political Violence," which resulted in nearly 300,000 deaths. After an armistice was reached between the two sides, referred to as the *Frente Nacional*, the homicide rate declined to a level of 20 per 100,000 inhabitants. However, at the beginning of the '80s, Colombia faced a rapid increase in violence. This time the increase was the result of cocaine trafficking and the rise of the guerrillas, and the homicide rate reached 80 per 100,000 ^6^ ^,^ ^11^.

The fact that Colombia had 16 homicides per 100,000 in 1938-a rate that would be considered high today-along with episodes of such high social violence suggests that the country indeed bears the social burden of violence produced by its multiple conflicts. The drastic and rapid fluctuations observed in Colombia's homicide rates bring to mind sociocultural phenomena such as political violence or drug trafficking more so than genetic mutations fluctuating over periods of dozens of years or more. A sociocultural component lying at the root of Colombian society may indeed facilitate the endemic explosions observed. 

The ACTIVA survey carried out by the CISALVA Institute (at the Universidad del Valle) in various cities throughout Colombia revealed a 47% approval rate for taking justice into one's own hands. Additionally, 47% believed in the right to kill in order to defend one's family, and 36% approved in some way of eliminating delinquents through so-called "social cleansing"^12^ ^,^ ^13^. Similarly, it was found that one-third of fathers and mothers who said they did not abuse their children responded affirmatively when asked if they hit them with a hard object that could hurt them. In Colombia, child abuse, with its components of physical punishment and emotional abuse, including negligence, is so common that it goes unnoticed.

The case of an 11-year-old girl detained by police after hiring an assassin to kill her mother because she abused her so badly, a story that appeared in Colombian newspapers, gives the impression that the seeds of violence are sown very early in Colombian homes.

Recent scientific findings have demonstrated that violence and child abuse in the first years of life are extremely damaging. Imaging studies of Romanian children that were the victims of extreme abuse and abandonment show reduced brain development, particularly in the pre-frontal cortex areas that manage specific behaviors in humans: long-term vision, impulse control, the ability to concentrate, planning, altruism and empathy ^14^. It can be postulated, then, that children born in violent environments who experience violence even in the womb and who later grew up in dysfunctional families become adults with a propensity to solve conflicts with violence. 

Literature from around the world has emphasized that child abuse has profound physical and mental consequences in adults and that child abuse prevention should be a basic strategy for preventing violence in society ^15^.

Colombia should pursue a profound cultural change, beginning from the very earliest stages of life, so that violence ceases to be a culturally accepted way of resolving conflicts. Only in this way can we foster a generation of Colombians who live in solidarity, are non-violent and are able to live in peace. 
