# Is Colombia a violent country?

**Published:** 2017-06-30

In this article by Guerrero and Fandiño-Losada, On page 10, Figure 1, x axis name is incorrect: "Rate x 1,000 K hab." The correct name should have read: "Rate x 100,000 hab."

