# Supplementary material for: Is Colombia a violent country?
Source: Colomb Med (Cali). 2017 Jun 30;48(2):101. (PMC5597094)
Supplement: Translation

# Translation: ¿Es Colombia un país violento?

Este artículo de Fandiño-Losada and Guerrero, aparece en la pagina 10, figura 1, el nombre del eje x es incorrecto: "tasa x 1,000 k hab.", el nombre correcto es "tasa x 100,000 hab."
